# Trisomy 22 Mosaicism from Prenatal to Postnatal Findings: A Case Series and Systematic Review of the Literature

**DOI:** 10.3390/genes15030346

**Published:** 2024-03-08

**Authors:** Valentina Trevisan, Anna Meroni, Chiara Leoni, Fabio Sirchia, Davide Politano, Giacomo Fiandrino, Valentina Giorgio, Donato Rigante, Domenico Limongelli, Lucrezia Perri, Elisabetta Sforza, Francesca Leonardi, Germana Viscogliosi, Ilaria Contaldo, Daniela Orteschi, Luca Proietti, Giuseppe Zampino, Roberta Onesimo

**Affiliations:** 1 Centre for Rare Diseases and Birth Defects, Department of Woman and Child Health and Public Health, Fondazione Policlinico Universitario “A. Gemelli” IRCCS, 00168 Rome, Italy; chiara.leoni@policlinicogemelli.it (C.L.); valentina.giorgio@policlinicogemelli.it (V.G.); domenico.limongelli@guest.policlinicogemelli.it (D.L.); lucrezia.perri@guest.policlinicogemelli.it (L.P.); germana.viscogliosi01@icatt.it (G.V.); giuseppe.zampino@unicatt.it (G.Z.); roberta.onesimo@policlinicogemelli.it (R.O.); 2 Genomic Medicine, Department of Life Sciences and Public Health, Catholic University of the Sacred Heart, 00168 Rome, Italy; francesca.leonardi@unicatt.it (F.L.); daniela.orteschi@policlinicogemelli.it (D.O.); 3 Human Genetics, Molecular Medicine Department, University of Pavia and IRCCS Fondazione Policlinico San Matteo, 27100 Pavia, Italy; anna.meroni01@ateneopv.it (A.M.); fabio.sirchia@unipv.it (F.S.); 4 Department of Brain and Behavioral Sciences, University of Pavia, IRCCS Mondino, 27100 Pavia, Italy; davide.politano01@universitadipavia.it; 5 Department of Molecular Medicine, Anatomic Pathology Unit, University of Pavia and Fondazione IRCCS San Matteo Hospital, 27100 Pavia, Italy; g.fiandrino@smatteo.pv.it; 6 Department of Life Sciences and Public Health, Catholic University of the Sacred Heart, 00168 Rome, Italy; donato.rigante@unicatt.it; 7 Child Neurology and Psychiatry Unit, Fondazione Policlinico Universitario “A. Gemelli” IRCCS, 00168 Rome, Italy; elisabetta.sforza@unicatt.it (E.S.); ilaria.contaldo@policlinicogemelli.it (I.C.); 8 Genetic Unit, Fondazione Policlinico Universitario “A. Gemelli” IRCCS, 00168 Rome, Italy; 9 Department of Orthopaedics and Traumatology, Fondazione Policlinico Universitario “A. Gemelli” IRCCS, 00168 Rome, Italy; luca.proietti@policlinicogemelli.it

**Keywords:** trisomy 22 mosaicism, mosaic aneuploidies, mosaic trisomy 22

## Abstract

Background: Among aneuploidies compatible with life, trisomy 22 mosaicism is extremely rare, and only about 25 postnatal and 18 prenatal cases have been described in the literature so far. The condition is mainly characterized by facial and body asymmetry, cardiac heart defects, facial dysmorphisms, growth failure, delayed puberty, and variable degrees of neurodevelopmental delay. Problem: The scattered information regarding the condition and the dearth of data on its natural history and developmental outcomes restrict genetic counseling, particularly in prenatal settings. Moreover, a prompt diagnosis is frequently delayed by the negative selection of trisomic cells in blood, with mosaicism percentage varying among tissues, which often entails the need for further testing. Purpose/topic: The aim of our work is to provide assistance in prenatal and postnatal genetic counseling by systematically delineating the current knowledge of the condition. This entails defining the prenatal and postnatal characteristics of the condition and presenting novel data from three cases, both prenatally and postnatally. Additionally, we report the developmental outcomes observed in two new patients.

## 1. Introduction

Full trisomy 22 ranks as one of the most frequently identified chromosomal abnormalities in spontaneous abortions, and is regarded as highly lethal during the neonatal period [1]. Mosaicism is defined as the existence of two or more cell lines in an individual, with distinct genotypes, all originating from a fertilized egg [2]. Mosaic trisomy 22 (mT22) is a rare aneuploidy which has prolonged survival compatibility [3]. The longest reported survival for an Individual with complete trisomy 22 is 3 years, whereas mosaic trisomy 22 allows for a more extended lifespan [4], with the oldest case of 19 years old being described in 2004 by Woods and colleagues [5].

The phenotype observed in individuals with mosaic chromosomal abnormalities is less severe compared to those with complete chromosomal aneuploidy, and the level of mosaicism can vary across different tissues [6]. Patients with mT22 might present a wide clinical spectrum characterized mainly by growth restrictions, webbed neck, microcephaly, facial anomalies, heart defects, limb malformations, dysmorphic features, and hemihyperplasia. Neurodevelopmental outcomes range from normal to severe intellectual delay [7,8].

In the case of suspected mT22, a prompt diagnosis is frequently hampered by the negative selection of trisomic cells in blood, low level of mosaicism, and the presence of uniparental disomy [9].

Nowadays, the postnatal phenotype of only 25 patients with this condition have been reported. Because of its rarity, the phenotype associated with mT22 is only partially delineated, and particularly little is known about the natural evolution of the condition and its neurodevelopmental outcome. Furthermore, the limited quantity of published data makes the prenatal genetic counseling complex, as little information can be provided to parents regarding the postnatal life.

With this review, we aim to provide a practical overview of Trisomy 22 mosaicism, including insights into the prenatal features, expected clinical progression of patients, and further delineation of neurodevelopmental aspects of the condition. Finally, we will use an overview of the current scientific research to try to explore the current and future directions of management.

## 2. Materials and Methods

**Figure 1:**
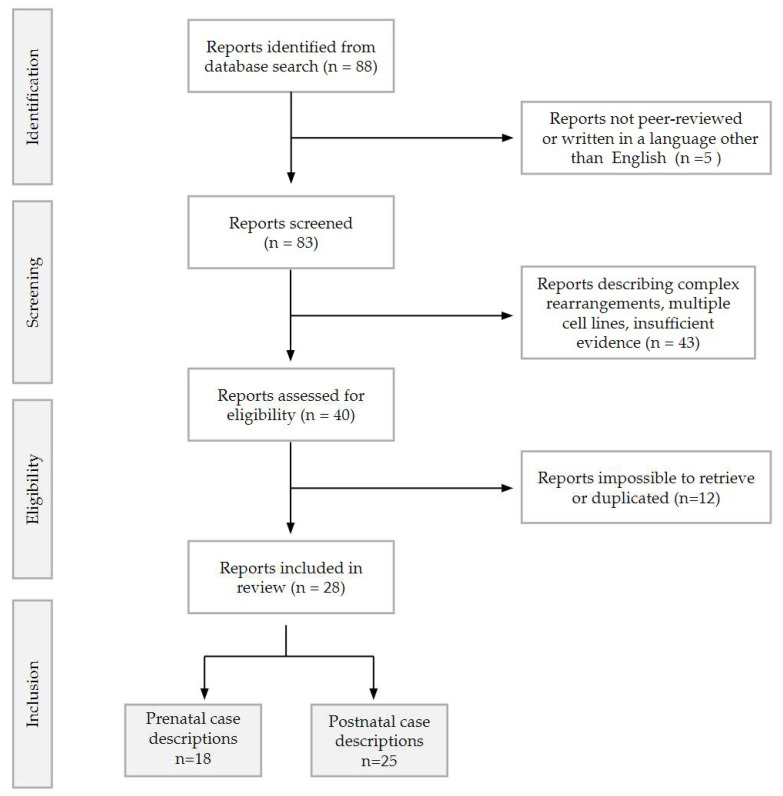
Flowchart describing the records selection for the review, as per PRISMA 2020 guidelines [10].

In this paper, we described a case series of prenatal and postnatal cases collected in two Italian Tertiary Hospitals (San Matteo Hospital IRCCS in Pavia and Fondazione Policlinico A. Gemelli—IRCCS), followed by a review of the literature. Bibliographic research was performed on all studies concerning trisomy 22 mosaicism until December 2023, following the PRISMA 2020 Guidelines [10]. A comprehensive online search was performed independently by two reviewers (V.T. and A.M.) in PubMed, MedlinePlus [11], and WebOfScience. Search terms included the following free-text terms: ‘trisomy 22’, ‘mosaic 22′ and ‘mosaicism 22′. An initial number of 88 papers was sought. After filtering the results limiting the search to human pregnancies in the English language, 83 articles were initially included. Among them, only peer-reviewed studies in English were selected, divided in prenatal and postnatal and then sorted by year of publication. Postnatal studies reporting patients with complex rearrangements [12,13,14] or describing multiple cell lines [15] or providing insufficient evidence [16] were excluded from the postnatal cohort, along with papers that were not possible to retrieve. All cases described both prenatally and postnatally were counted once, leading to a final number of 28 articles being included, reporting a total of 18 prenatal and 25 postnatal cases. A flowchart of the reports selection process is described in Figure 1. A summary of the prenatal findings is presented in Table 1 and Table 2, while postnatal findings are described in Table 3.

Moreover, we included in the study three additional individuals with mT22: one for the prenatal cohort (case A), one for the postnatal group (case C), and the latter encompassing both categories (case B). The newly reported cases are described below.

### 2.1. Prenatal Case A

A 41-year-old primigravida came to our attention at 16 weeks of gestation. She performed the combined first trimester screening for common trisomies [24] in another hospital, with a calculated risk of 1:13 for trisomy 21. BetaHCG levels were 2.88 MoMs, PAPP-A level was 0.23 MoMs, and the Nuchal translucency (NT) was remarkably normal, with a measurement of 1.3 mm [25]. The patient was offered the option of an invasive test, but she opted for non-invasive prenatal testing (NIPT) instead, with a low risk for trisomies 13, 18, and 21, but a high risk for trisomy 22, and a fetal fraction of 9% (Harmony prenatal test, Ariosa Diagnostics (San Jose, CA, USA)). At the ultrasound scan at 16.5 gestational weeks, fetal biometry was harmonically below the 5th centile for the gestational age [26], with oligohydramnios. All upper and lower limbs were fixed in a flexed position. At the brain level, the ventricles seemed prominent, without any early sign of the formation of cavum septi pellucidi. The cerebellum was of normal size, but the cisterna magna was completely obliterated, and vermis could not be assessed in the absence of a detectable spinal defect. A dysmorphic fetal profile with micrognathia with a thick nuchal fold was noted. At the cardiac level, a suspicion of double-outlet right ventricle was raised. A single umbilical artery was also present, and external genitalia were feminine. A multidisciplinary counseling with an obstetrician and geneticist was organized, and the patient underwent amniocentesis at 17 gestational weeks, which resulted in mT22 on amniotic fluid; 47,XX,+22[5]/46,XX[25] on 4 cultures. The fetus died in utero at around 19 weeks of gestation. At the post-mortem examination, dysmorphic traits were described, such as frontal bossing, flat nose, up-slanting eyelids, wide mouth, micro-retrognathia, and low set ears with dysmorphic helices. A thick nuchal fold was confirmed; the thymus was present but hypoplastic, with signs of lymphocytic depletion at the microscopic level. Hands were bilaterally flexed ventrally on the wrists, and the fingers of the left hand were laterally deviated. Left talipes varus was present, and muscle hypotrophy was especially evident in the lower limbs. The umbilical cord had a single umbilical artery, and was extremely hyperspiralized, with numerous reductions of caliper. At the placental level, anomalous morphology of the chorion villi was observed, with trophoblastic hypertrophy and gravidic decidual arteriopathy with acute atherosis.

### 2.2. Prenatal and Postnatal Case B

A 31-year-old secundigravida unipara came for a first trimester scan in a spontaneous monochorionic diamniotic twin pregnancy. The combined screening for trisomies [24] resulted in a high risk for trisomy 21 in one of the twins (1:81, with NT 3.6 mm, free BetaHCG 1.96 MoM and PAPP-A 1.77 MoM). At 16 weeks of gestation, an amniocentesis was performed on both amniotic sacs. Fetus “A” showed a normal karyotype (46,XY), while fetus “B”, with a high risk for aneuploidies, proved to be mT22 with karyotype showing 47,XY+22[4]/46,XY[21] on 3 cultures. The presence of persistent left superior vena cava (pLSVC) was noted at the anomaly scan, in the absence of other cardiac or extracardiac anomalies. The fetuses grew regularly until the end of the second trimester. The mother developed gestational diabetes mellitus, which required insulin, and also developed gestational hypertension in the third trimester, when the twin with mT22 started not to gain weight and the discrepancy between the twins’ weight became 19%. A cesarean section was performed at 33.6 gestational weeks for preeclampsia in the woman with a twin pregnancy and who had a previous cesarean section. The weight at birth for the baby with mT22 was 1705 g (11th centile for the gestational age [27], with a total length of 41.5 and a cranial circumference of 31.5 cm. Apgar scores were 6 at one minute and 8 at 5 min of life. At the pathology observation, the presence of two distinct placental discs suggested dizygosity of the twins. The placental district of the mT22 twin presented immature chorionic villi with signs of chorangiosis, characterized by clusters of avascular villi or villi with signs of vascular-stromal karyorrhexis, as in fetal vascular malperfusion. The placenta was also characterized by intra- and perivillous fibrin deposits, with calcifications and areas of infarction. After delivery, the newborn was hospitalized for respiratory distress syndrome and prematurity. After birth, hypospadias was diagnosed, which required surgical correction. No further major malformation was reported. Cardiologic follow-up was carried out for the pLSVC for the first two years of life, and no complications, for example aortic coarctation, were registered. The urological and brain ultrasounds were normal. The child was longitudinally followed, and growth parameters were reported between the 10th and 50th percentile. Besides severe epicanthus and suspected strabismus, no major dysmorphism was documented.

At 8 months and one year and ½ of life, the growth was constant with the following measurements for a corrected age of 18 months: height was 76 cm, below the 10th centile for the age; weight was 10.3 kg (<10° pc); and OFC 49 cm (50° pc). The child did not present any major neurological issues at the time, with developmental milestones considered adequate for age and prematurity.

### 2.3. Postnatal Case C

A 17-year-old boy was referred with mT22 for clinical follow-up. Pregnancy was characterized by IUGR and reduced placenta function. CVS sampling was performed formaternal age and prenatal features identifying mT22 (47,XY+22[95]/46, XY[5]), while blood karyotype performed from the umbilical cord at birth turned out normal (46,XY). Therefore, genetic investigations performed so far showed a homogeneous T22 mosaicism apparently confined to the placenta, with other tissues yet to be analyzed. At birth, besides hypotonia, major dysmorphisms were noticed at birth, including right eye ptosis and a webbed neck. Echocardiography revealed ASD associated with moderate left to right shunt and persistence of superior vena cava in the coronary sinus. Persistence of the brachiocephalic vein was initially suspected and then confirmed. Congenital anomalies identified at birth included an anteriorized imperforated anal orifice, urogenital anomalies including cryptorchidism, fan shaped foreskin, and postural bilateral talipes, corrected by manipulation. Audiological screening and the fundoscopic exam turned out normal. Concomitantly with anal corrective surgery, a skin biopsy was performed, which confirmed 3% mT22 mosaicism. The result was confirmed by further skin biopsy performed at our institution (13% mT22 mosaicism). The patient also underwent surgery for blepharoptosis and bilateral cryptorchidism. Growing severe kyphosis was documented (Scheurman disease), which required a neurosurgical approach two distinct times. His growth pattern was harmonic but below the standard with head, height, and weight around 10th, 4th, and 2nd percentile for age, respectively [28]. At 17 years of age, he developed secondary sexual characteristics such as a beard, even though his voice’s pitch remained nasal. He presented the following peculiar facial characteristics: high forehead, midface hypoplasia, partial ptosis, epicanthal folds, sparse eyebrows on the lateral third, long beaked nose, webbed neck, protruding ears with simplified helix, and a high arched palate with dental crowding. Fingers seemed long and slender with bilateral clinodactyly. Despite multiple spinal surgeries, kyphosis was still noticeable. An MRI performed at one year of age confirmed the absence of macroscopic malformations. As a secondary finding, white matter hyperintensity in periventricular space was associated with dilated perivascular spaces related to terminal areas of myelination. Signals of incomplete myelination was also detected in the subcortical U fibers. Neurodevelopment was mildly delayed, and the cognitive profile at 14 years of age showed a borderline result QIT 81 (WISC-IV). He attended high school with a support teacher, and he was independent in self-care. During the last follow-up, parents noticed behavioral challenges, including oppositional behavior and difficulties in social interactions.

## 3. Results

### 3.1. Prenatal Phenotype: General Overview

Trisomy 22, in its complete or mosaic form, has an estimated incidence of up to 3–5% in spontaneous abortions [29]. While complete trisomy 22 is often non-compatible with life, leading to death in utero or shortly after birth [30], mosaic forms may survive through pregnancy and thrive in the postnatal life.

To date, including our new case reports, 20 cases of prenatal mosaic trisomy 22 have been reported after a diagnosis was carried out through chorionic villi sampling and/or amniocentesis (see Table 1). Among these cases, four did not have any abnormal morphologic feature [1,17,18], while 16 cases were instead characterized by abnormal findings at the scan. Female preponderance was striking, with 14 female cases versus 6 male cases. Amongst the reported cases, the level of mosaicism for trisomy 22 in the amniotic fluid cells was between 0% in the cases of confined placental mosaicism [18,20,23] and 60% [22]. The highest mosaic percentage in amniotic fluid in a liveborn was 35% [21]. The preferred method of postnatal cytogenetic confirmation was peripheral blood (11 cases), registering the highest percentage of mosaicism in the case described by Schinzel et al. (90%, case of Intrauterine Death in the third trimester) [3]. The second preferred confirmation method was skin biopsy. In our cases, postnatal karyotype confirmation was not carried out due to parental will. Placental mosaicism was present both in the cases with a poor obstetric outcome (up to 100% of trisomic placental cells, perinatal death at 24 gestational weeks in the case series of Minella and colleagues [23]) and in the cases with a good outcome, such as for the patient described by De Pater and al. (liveborn at 39.6 gestational weeks) [9]. Uniparental disomy (UPD) was tested in five cases, where two were maternal, and three cases showed chromosomes originating from both parents. Depending on the mosaicism grade, the prenatal phenotype ranged from normal fetuses tested for maternal age only [1], to IUGR without any detectable morphological abnormality in the cases of confined placental mosaicism [17,23], to a vast number of fetal malformations.

#### 3.1.1. Intrauterine Growth Restriction (IUGR)

IUGR is the most common characteristic of fetuses with mT22. It is described in almost all of the prenatal case reports, even if the severity and the pattern of the growth retardation differ. Some fetuses might be small for gestational age early in pregnancy [7,22,23], while in other cases, only a late growth restriction is present [6,9,17]. These differences can probably be attributed to different mosaicism grades in the placental tissue, placental aneuploidy being a known cause of growth retardation [31]. An impaired placental function might also be the cause of maternal preeclampsia [23] and of oligohydramnios in some of the presented cases [22]. Interestingly, the latest reported cases from Minella et al. describe the placenta hormonal values for the combined screening for trisomies in the first trimester [32], showing decreased levels of PAPP-A (0.24 MoMs, 0.19 MoMs and 0.17 MoMs respectively), increased levels of BetaHCG (7 MoMs, 1.99 MoMs and 10.57 MoMs respectively), and normal nuchal translucency [23].

#### 3.1.2. Increased Nuchal Translucency (NT) in the First Trimester

**Figure 2:**
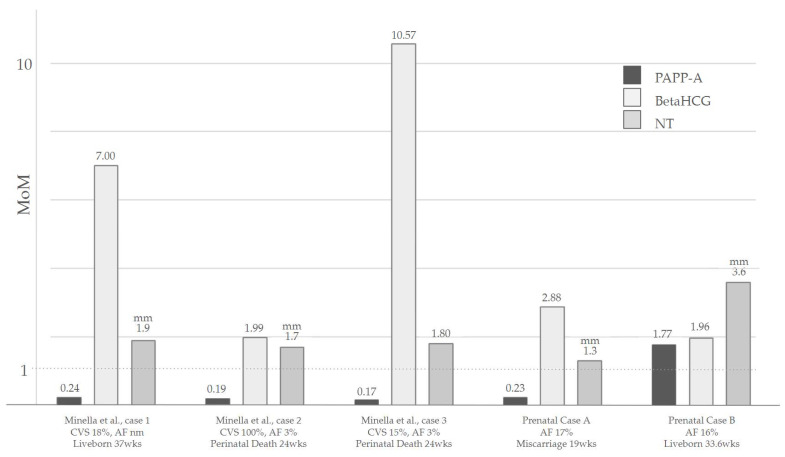
A summary of the NT values and the placental hormones in the first trimester of the Minella et al. cases [20] and our cases. Abbreviations: CVS: Chorionic villi sampling; AF: Amniotic fluid; wks: Weeks; NT: Nuchal translucency. PAPP-A and β HCG are reported in MoMs, while NT is expressed in [mm].

An increased NT at the first trimester scan is a known marker for aneuploidy, widely known for its high prevalence in trisomy 21 [33,34]. For cases of mT22, first trimester NT could be described either in the normal range [23] or above the normality thresholds for the gestational week [7,21]. In our cases, NT was increased in the patient of Prenatal Case B, while it was in the normal range for Case A. As for other aneuploidies and genetic syndromes, its presence can be related to an impairment of the lymphatic system, to the underlying presence of a cardiac defect, or to an early systemic impairment [35]. An increased nuchal fold is described to persist at the anomaly scan in the second trimester, and may result in the presence of a Turner-like webbed neck also reported in a few postnatal cases [7,21]. A summary of the NT of the Minella et al. cases and our cases is shown in Figure 2.

#### 3.1.3. Other Reported Prenatal Abnormalities

Unspecified abnormal findings were reported in a male fetus who unfortunately experienced intrauterine death at 33 weeks [1], while in another fetus from the same case series, hydrothorax was reported [1], and it was associated with increased nuchal fold. Berghella and colleagues described a fetus with mT22 and the presence of a choroid plexus cyst [19], which is known in literature to correlate with genetic syndromes in some cases [36]. Another soft marker for aneuploidies, the single umbilical artery, was described in one case by Abdelgadir and colleagues [7]. A short femur was reported in two cases where overall IUGR was also noted, probably denoting a primitive rhizomelic bone growth insufficiency rather than fetal growth restriction of placental origin [7,23]. In our Prenatal Case A, the pathologic examination reported a hypoplastic thymus, the presence of talipes, flexed hands, and limbs in a fixed position.

### 3.2. Systematic Review of the Postnatal Phenotype and Shared Features between Prenatal and Postnatal Phenotype

According to our knowledge, 25 cases of mT22 have been described in the literature so far (Table 3). The female to male ratio observed in the prenatal cohort was less striking in the postnatal cohort, with 60% of cases being genotypically female (see Table 3). Mosaic trisomy 22 represents a rare chromosomal anomaly syndrome characterized by a highly variable phenotype. Patients might exhibit characteristics of full trisomy 22, resembling Turner-like stigmata, or present with a less obvious clinical picture of the condition [1,7].

The following paragraphs will highlight the key clinical characteristics documented in the literature, starting with those shared between the prenatal and postnatal phenotype, and subsequently addressing those specifically associated with the postnatal phenotype.

### 3.3. Craniofacial Dysmorphic Features

Phenotypically, almost every individual displayed some dysmorphic features, both in the prenatal and in the postnatal series. Among the various dysmorphisms reported, only those cited in >10% were considered distinctive and therefore mentioned. The most frequent organ involved is the ear, with four prenatal cases of low-set ears described in post-mortem reports [19,22,23,37], with or without external ear anomalies, such as a crumpled helix [37]. In the postnatal cohort, ears were low-set in 11 cases out of 25, often accompanied by anomalies (10/25) and almost constantly associated with pre-auricolar tags or pits (14/23). After birth, the head might be microcephalic (6/25), characterized by frontal bossing (8/25). Eyes were characterized by hypertelorism (10/25), epicanthal folds (9/25), and unilateral or bilateral ptosis (6/25). Hypertelorism was also described in four prenatal cases [19,21,22,23,31,37]. A broad flattened nasal bridge (9/25) with a long smooth philtrum (5/25) and anteverted nostrils (5/25) was distinctive. Lips were occasionally reported as thin (3/25). Retrognathia and micrognathia are common in fetuses and children with mT22, being reported in seven cases of the postnatal cohort and prenatally by Schinzel et al. and Minella et al. [23,37]. It is not always clear whether they are an isolated malformation of the temporo-mandibular bones or an adaptive change to the presence of neuromuscular issues. The neck commonly had a short and webbed appearance (6/25), marked by a low posterior hairline (7/25). Evident craniofacial asymmetry was also present in 32% of cases (8/25) commonly associated with midface hypoplasia in 5/25 of the individuals.

A true midline facial cleft was reported in one prenatal case only, by Chen and colleagues [22]. General palate clefting was reported in four cases overall [1,38,39,40], in one instance affecting only the soft palate [40].

### 3.4. Cardiovascular Malformations

Congenital heart diseases represent a prevalent factor in pediatric mortality, affecting eight out of every 1000 live births. Among congenital heart diseases, the atrial septal defect (ASD) manifests in 7−11% of cases. In general, up to 33−42% of congenital heart diseases are linked to chromosomal aneuploidy [41], and up to 50% of patients with mT22 have been reported with cardiac defects [21]. Various types of cardiac abnormalities are reported in prenatally-detected mT22. The most commonly described feature prenatally is ASD [3,6,7,21], although its diagnosis has been made mostly after birth, confirming the well-known difficulties in the detection of ASDs in prenatal life [42]. Other cardiovascular anomalies detected at the prenatal ultrasound were an isolated perimembranous ventricular septal defect (VSD) [9] and aberrant right subclavian artery (ARSA), described prenatally in two cases, but never as an isolated finding [3,23]. Tetralogy of Fallot (TOF) has been reported in one case prenatally and two cases postnatally: Once, in a female fetus who unfortunately underwent perinatal death at 24 gestational weeks [23], and postnatally in one boy and one girl as part of a complex heart defect which will be further detailed later [7,40]. Prenatal detection of hypoplasia of the right ventricle with tricuspid atresia has also been described [37], while some cases, on the contrary, describe a right ventricular preponderance [23]. Nevertheless, the finding in this last case coexisted with the presence of IUGR with vascular impairment, and could be therefore not a malformative, but an adaptive finding [43]. Finally, prenatal ultrasound also detected one patient with VSD and a hypoplastic transverse aortic arch [40]. Details regarding postnatal cardiovascular features of mT22 are more comprehensive and heterogeneous.

According to the literature, cardiac abnormalities affect 65% of postnatal mT22 patients (Table 3), ranging from isolated to complex cardiac defects, including a combination of ASD, VSD, pulmonary stenosis (PS), and patent ductus arteriosus (PDA) to [5,6,7,8,9,39,44], as well as malformations, like TOF [7,23]. Interestingly, evidence shows that complex heart defects or combined defects, defined as the presence of a congenital heart defect associated with a vascular anomaly or a structural heart defect, were more prevalent (9/25) than isolated ones (5/25). In detail, data indicate that approximately 28% of mT22 patients present ASD, and in every instance, this defect was part of a more complex cardiovascular presentation. On the other hand, VSD is present in 24% of patients, and was reported as an isolated defect in two distinct cases [5,9]. PS, detected in 16% of all mT22 cases, was reported as severe in two separate individuals who required balloon-plasty [7,8], mild in one case as an isolated defect [45], and of unspecified entity in Fruhman et al. [46]. The Tricuspid was the second valve most commonly involved, with three cases exhibiting abnormalities of the anatomical valve as part of a complex heart defect that will be described in the following sections. Tricuspid valve defects annotated were as follows: moderate tricuspid regurgitation [7], tricuspid atresia [37], and tricuspid stenosis [46]. PDA was less common, affecting three patients in the whole review cohort [7,47], with one case requiring surgical correction [7]. Interestingly, non-compaction of the ventricles has been observed in two postanal cases. The first patient was initially described by Wang et al. and later reported also by Abdelgadir et al. [7]. Defects identified were LVNC and the presence of a ductal aneurysm, which led to progressive PS, and had to be treated with ballooning after birth [21]. In the second patient, left ventricular non compaction was identified as an isolated finding [48]. Complex heart defects were reported in five cases [3,7,46,47]. Schinzel et al. described a patient with VSD, ASD, tricuspid atresia, and a retroesophageal position of the left subclavian artery [37]. Pridjian et al. reported an individual with ASD malformation, PDA, and PS [47]. Fruhman G et al. documented a case characterized by ASD, TOF, PS, tricuspid stenosis, and an hypoplastic hypertrophied right ventricle [37,46]. Finally, the two patients reported by Abdelgadir et al. showed complex cardiac defects: one exhibited a complex heart defect characterized by an ASD which spontaneously closed at 2 years of age, PDA, severe PS, moderate tricuspid regurgitation, and LVNC, while the second had TOF, PDA requiring surgical correction, mild residual left pulmonary artery hypoplasia, and mild right ventricular volume loading [7]. In this last patient, a heart biopsy was performed during the surgical corrective operation of the cardiac defects, revealing a 17% mosaicism for mT22 for the cardiomyocytes [7]. Combined defects were described in 16% of the patients (4/25). In detail, two patients exhibited ASD and persistence of superior vena cava in the coronary sinus [6,39]. One patient had ASD along with hypoplasia of the pulmonary branches without hemodynamic consequences [44]; another showed VSD and a hypoplastic transverse aortic arch [40]. It is noteworthy to mention that malformations affecting the cardiovascular system extend beyond the heart structure to include great vessels. Hypoplasia of the pulmonary branches have also been reported in two cases [44], also causing mild right ventricular overloading [7]. A retro-esophageal position of the left subclavian artery [3], double aortic arch, and an aberrant subclavian artery anatomy [12]. For small vessel anomalies, hemangiomas were reported in two cases [12,21], one with a spontaneous resolution [7]. Given the prevalence and the complexity of the cardiovascular defects, it is recommended to include a meticulous cardiovascular assessment encompassing a cardiovascular visit, echocardiography, and electrocardiogram (ECG) as part of multidisciplinary management.

### 3.5. Urogenital Findings and Puberty

Renal anomalies have been detected in three patients: one pelvic kidney [12], one anterior-facing renal pelvis with dilated ureters [46], and one patient with signs of hydronephrosis [39,49]. Definitive data about puberty and fertility in patients with mT22 are scarce and fragmented. According to the literature, abnormalities of external and internal genitalia emerged in 40% of males (6/25), apparently slightly more affected than females (4/25). Among female patients, primary ovarian failure has been reported in four patients. One patient presented normal sized ovaries and no pubertal development [12], while another patient has been reported with ovarian hypofunction [49]. Two female patients have been reported with internal genital anomalies, such as the absence of one ovary and fallopian tube [12], or a unicornuate uterus with a normal round ligament, along with a fallopian tube and a streak ovary [5] with absent pubertal development. External genital anomalies, showing as a hypoplastic labia majora, were observed in two patients [3,21]. Among male patients, no documentation of puberty in male patients were discovered. Cryptorchidism, either unilateral or bilateral, was the external genital anomaly most frequently observed, in 4/23 patients [6,39,44,47], while just in one case, a testicle was reported to be descended but small in size with respect to development [50]. As an external anomaly, hypospadias was reported in three cases: in one case isolated [51], in one case associated with cryptorchidism [6], and in another case along with ambiguous genitalia [39]. Prenatally, it was encountered in one case only [20]. To our knowledge, information about the reproductive possibility of patients with mT22 is not available. External anomalies are present in both sexes, suggesting some sort of endocrine dysfunction which has not been proven so far. It is therefore advisable to exercise attention in counseling parents regarding mT22 reproductive potential.

### 3.6. Ophthalmic Manifestations

Ptosis, affecting around 24% of patients, is a quite remarkable sign of mT22. It was reported in four out of six cases as unilateral [39,46,48]. Moreover, a heterogeneous range of ophthalmologic anomalies were detected in 36% of patients since birth. One patient was reported with bilateral glaucoma [1]. Coloboma was reported in two cases: in one case, it was a choroidal coloboma involving the macula [48], while in the other case, it was bilateral involving the iris [46]. Strabismus was reported in two cases [48,49], while esotropia has been reported in two cases [39,48]. Anomalies of the iris, such as asymmetric irises [49] or scalloped pupils [47] were also reported in two distinct cases. Refractive errors, such as myopia and astigmatism [8,48] have also been reported. Our Case C needed ptosis surgical correction and showed astigmatism, while Case B might develop refractive errors with aging. In total, 43% of mT22 patients experienced some degree of ocular compromise since birth. It is therefore advisable to include longitudinal ophthalmologic monitoring as part of multidisciplinary management to address potential neurovisual impairment.

### 3.7. Ears and Auditory Apparatus

Since the initial publications, ear anomalies have been identified as a distinctive sign of mT22 [37,52]. Ears are commonly reported dysmorphic (40%) and low-set (44%) unilaterally or bilaterally. They are typically described as protruding and posteriorly rotated [44,47,53], with simplified or crumpled helices [7], or with hypoplasia of the antitragus, tragus, and lower helix [39]. Ear anomalies were almost invariably associated with pre-auricular tags or pits, and just in one case, a skin tag was also present [46]. Stenosis of the external auditory meatus was reported in just two cases [5,54]. Just one case was reported to have the Goldenhar sequence [47]. Even though more data are needed to define the type of hearing loss and the exact timing of onset, the actual evidence suggests that 24% of mT22 subjects (6/25) develop bilateral or unilateral hearing loss for still unexplained reasons. Therefore, a thorough otorhinolaryngologist assessment along with a longitudinal audiometric evaluation should be offered to all mT22 patients [44].

### 3.8. Growth Parameters

In up to 60% of cases, the growth pattern has been reported as delayed, with 32% of patients showing failure to thrive. Among these, one subject required nasogastric tube feeding [44] and another gastrostomy due to a severe gastro-esophageal reflux [40,49]. We recommend using this information judiciously, since growth outcomes of these patients have not been reported. For instance, our Case B does not seem to show any growth abnormalities, while the late adolescent patient showed satisfactory growth. Taking this into account, for mT22 patients, data suggests to closely monitor the growth pattern and rule out possible gastrointestinal diseases (i.e., GERD) [40] or any endocrine disorders throughout childhood [39].

### 3.9. Neuropsychiatric Features

Intellectual disability (ID) is defined by DSM-5 as a defect in intellectual functioning and adaptive behavior with onset in the developmental period that influences the conceptual, social, and practical domain in daily life [55]. Currently, in clinical settings, adaptive functioning is the primary determinant of the severity of intellectual disability (ID), whereas in research contexts, greater emphasis is placed on IQ measurements. To accurately delineate the severity of ID in most patients, it is essential to assess both adaptive functioning and IQ [56]. Approximately 2–3% of the worldwide population is affected by ID (IQ < 70) [57]. Due to ID’s genetically heterogeneous nature, a genetic basis can be quite challenging to reach. Notwithstanding the improvements in diagnostic technologies, when suspecting mosaic imbalances, classic karyotyping still has an impact on diagnosing genetic ID. According to our review, neurocognitive development information was available in 12 of the 25 patients. Among these, three patients showed normal intellectual functioning, five had mild ID, three had moderate ID, and one had severe ID (Table 3). Various degrees of developmental delay have been reported in 6 out of 25, without a corresponding formal IQ evaluation. Interestingly, testing in two cases was not performed, since subjects were reported as meeting the standards [7,8], suggesting that there might be some potential improvement with aging, even if this should be proven through an objective assessment. Hypotonia was quite a common finding, detected in around 32% of the subjects (8/25 cases). Regrettably, data provided did not specify whether hypotonia was temporary or persisted with aging. Based on early descriptions, mT22 was consistently linked to ID, ranging from mild to severe [52]. However, the recent broadening of the understanding of the neurodevelopmental aspects of the condition suggests that the absence of ID could be a potential outcome for mT22 patients [7,8]. This consideration might be due to the progressive improvements in diagnosing even milder cases, or to a neurodevelopmental catch-up after an initial period of developmental stagnation. To further support this assumption, seven of the reported patients, including our patients of 17 years and 5 years, display a developmental outcome ranging from borderline to normal (Table 3). A previous study presented by Florez and Lacassie in 2005 showed that there was no apparent correlation between the proportion of trisomic cells and the severity of developmental delay [8]. However, it is worth mentioning that besides a longitudinal assessment performed by Crowe et al., most data reported refer to patients at relatively young age, which may mask other developmental issues or progress in school years [50]. Moreover, there is a lack of data concerning academic performance, potential learning disabilities, and supportive therapy such as speech therapy or the use of supportive learning tools. Therefore, this skewed observation might produce inaccurate predictions about the natural neurodevelopmental evolution of the condition. In order to precisely define the neurodevelopmental aspects of mT22, further descriptions of academic and neurodevelopmental progress in school years, adolescence, and adulthood are needed [7]. Since accurate information is critical for genetic counseling, we therefore suggest caution in predicting possible neurodevelopmental outcomes, especially in prenatal settings. Finally, a quite exceptional finding was seizures, reported just in one case during delivery, concomitant to cardiac arrest [39].

Central nervous system abnormalities were detected in three cases [1,7,47]. Pridjian and colleagues reported a male patient without a corpus callosum and a septum pellucidum with an enlarged 3rd and 4th ventricle [47], while a brain and spine MRI in Dalal Abdelgadir et al.’s study [7] showed prominent perivascular spaces, old microhemorrhages, and a tethered cord ending at L2L3. Finally, Leclercq et al. reported a patient with cortical atrophy [1].

### 3.10. Skeletal Anomalies

Around 52% of mT22 cases present body asymmetry [40,50], which predominantly manifest as hemiatrophy [21], uneven legs’ length [12,44,49,58], or one hand being noticeably smaller than the other [12,58]. As a consequence, scoliosis is a common finding as well [3,12]. Rib cage anomalies were less commonly noticed (4/23), with three cases of pectus excavatum [7,8] and one carinatum [52]. Cutaneous or partial syndactyly along with 5th finger clinodactyly were distal limb anomalies more commonly reported, in 28% (7/25) and in 44% (11/25), respectively, of postnatal cases, and in two of the prenatal cases [9,23,37,58]. In contrast with previous reports, cubitus valgus and foot anomalies such as talipes equinovarus requiring surgery [5], and hallux varus were seldom reported [12]. Among major skeletal deficiencies, terminal transverse limb reduction [44], thumbs hypoplasia [3], and agenesis of the distal phalanges of the left hand and foot [1] have been also reported in one case each, respectively.

Unprecedentedly documented, vertebral anomalies were also reported in 16% of cases (4/25). Cervical vertebrae were affected in two cases: one showing C2–C3 synostosis [1,46], and another patient with abnormal cervical vertebra C1 [12]. A mid-thoracic vertebral segmentation defect was detected in just one patient [1,46]. Altered bone maturation with coronal clefts from T1 to L3 was also detected [44]. Remarkably, our Case C presented Scheuermann disease, which severely affected his quality of life and required surgical correction in two distinct surgeries.

The following clinical features were observed exclusively in postnatal cases.

### 3.11. Skin and Nail Anomalies

From the initial characterization of mT22, ectodermal anomalies have consistently stood out as a distinctive feature. Review of literature data confirms this observation, with nail dysplasia and hypoplasia being the most common finding in 12/25 cases (48%) (Table 3). Nails might be spoon shaped or dysplastic, with abnormalities affecting both fingernails and toenails.

Pigmentary anomalies, whether characterized by excessive or insufficient pigmentation, following the Blaschko lines and associated to asymmetry in the body, are indicative of a mosaic genetic disorder [40]. Around 32% of mT22 patients showed pigmentary changes either along Blaschko lines [5,44,58], hypomelanosis of Ito [50], hyperpigmented changes [5,7,12,44], or hypopigmented ones [48,50]. Dental anomalies such as malposition, delayed dentition, aplasia, and notched incisors have also been reported in 24% of the cases (6/25) [5,50].

### 3.12. Gastrointestinal Tract Anomalies

Gastrointestinal tract abnormalities have been documented in association with various types of chromosome 22 alterations [59]. Gastrointestinal malrotation was reported in two cases. The first patient was described with a cecum in the right upper quadrant [12]. Then, Hall et al. reported a male newborn, diagnosed prenatally with de novo mT22, who had total colonic aganglionosis (TCA) and underwent a laparotomy for the correction of malrotation and midgut volvulus on the third day of life [54]. While a definitive causal link between TCA, malrotation, and mT22 has not been established, it is reasonable to think that malrotation and TCA share a common ground [54]. Biliary atresia (BA) or choledochal malformations are rarely caused by an underlying genetic abnormality [60]. A case series of five infants affected by various forms of chromosome 22 aneuploidies along with congenital biliary tract anomalies has been reported. Among them, one patient had trisomy 22 mosaicism and BA [51]. Given the report of Gangbo and colleagues of a 24-week gestation fetus with non-mosaic trisomy 22 without the gallbladder in autopsy, evidence seems to indicate that chromosome 22 may contain overlooked genes associated with early bile duct development [61]. On the other hand, anomalies of the anal region have been frequently reported. In our cohort, 16% of the patients (4/25) present some kind of anal anomaly, with 50% of them presenting anteriorized anus [6,7], while the other 50% present imperforate anus [3,46]. Also, our Case C presented with anal orifice malformation, which required surgical intervention. Based on the evidence provided, it is therefore advisable to carefully assess the potential presence of gastrointestinal anomalies in mT22.

### 3.13. Other Reported Abnormalities

As miscellaneous findings, dimples were also reported in four cases. Three dimples were observed in the sacral region [9,50], in one case associated with a pilonidal dimple [8]. The fourth one was an elbow dimple reported by Crowe at al A [44,50]. One case of mosaic trisomy 22 has been reported to be associated with increased larynx wall thickness, identified at laryngoscopy [39]. A miscellaneous report of BASM (polysplenia, absent vena cava, abnormal accessory portal vein, and nonrotation of the gut) was reported in the patient previously mentioned by Allotey J et al. [51]. Spleen absence was detected in just one case by Abdelgadir and colleagues [7].

## 4. Molecular Diagnosis in Prenatal and Postnatal Settings

Mosaicism occurs because of errors in fetal mitotic division or due to the mechanism of trisomy rescue, which may also result in maternal or paternal uniparental disomy. The diagnosis of mT22 might be complicated by the potentially low percentage of mosaicism in the tested cell lines, and by the presence or absence of mosaicism among different tissues [62]. Investigations for UPD for chromosome 22 have been inconsistent, as the impact of imprinting on this chromosome is considered to be minimal [20,63]. Conventional karyotype analysis on cultured blood lymphocytes could underestimate the percentage of mosaic cells due to selective disadvantages for the cells carrying an extra chromosome copy [64,65,66]. The prevailing hypothesis suggests that more robust selective pressures against abnormal cell lines (i.e., mT22) may occur in actively dividing marrow precursor cell lines, as opposed to ectoderm precursor cell lines [19]. In prenatal settings, differentiating confined placental mosaicism (CPM) from true fetal involvement is of fundamental importance [1,23]. According to our literature review, almost all the prenatally diagnosed patients with mT22 had confirmatory cytogenetic studies in postnatal life. Amongst the reported cases, the level of mosaicism for trisomy 22 in the amniotic fluid cells varied widely (0–60%) [18,20,22,23]. Placental mosaicism grade did not always correlate with the severity of the clinical picture. Minella et al.’s case series illustrates the complexity of antenatal diagnosis and the utility of both Array-CGH and FISH analysis on “direct” uncultured cells to avoid potential errors related to cell culture bias [23]. The lack of detection of trisomic cells at amniocentesis could be due to negative selection of trisomy 22 cells in culture and might lead to an erroneous diagnosis of confined placental trisomy. Therefore, the utility of the analysis of “direct” uncultured cells as a first diagnostic step should not be underestimated, either by interphase FISH, “direct” villus karyotype, or Array-CGH, considering the limited power of this technique in detecting low level mosaicism (<10–20%, depending on experimental conditions)”. The use of uncultured cells for the analysis permits an accurate genetic diagnosis and serves as a guide to appropriate counseling and management. Furthermore, requiring a shorter time than the analysis of cultured cells, it potentially allows a faster response to expecting parents.

Even if normal developmental outcomes have been reported, further data are needed to define the expected postnatal neurodevelopmental phenotype more precisely. Moreover, given that the trisomic cell distribution in fetal tissues cannot be predicted, parents should be aware that normal ultrasound findings do not necessarily indicate the absence of compromised psychomotor development in postnatal life.

Moreover, Array-CGH performed on DNA extracted from uncultured blood samples has been advocated to be superior to the sole karyotype in detecting the level of mosaicism [65]. For postnatal cases, an easy and increasingly popular procedure is to acquire buccal swabs, which require minimal invasiveness and patient distress. According to evidence, buccal swabs offer an increased probability of detecting cellular mosaicism, as several studies reported higher prevalence of trisomic cells in the saliva [64,67]. In cases where mosaicism cannot be identified in peripheral blood samples nor saliva, the analysis of skin fibroblast should be considered, as this cell type shows a higher prevalence of trisomy 22 mosaicism in some case series [7].

Concerning postnatal cases, based on our review, just 32% of the patients showed evidence of mosaicism in their blood, while it was almost invariably detectable as mosaic karyotype in skin fibroblasts. This data validates further previous findings of Abdelgadir et al. [7]. Moreover, just in three cases, peripheral blood karyotyping analysis was considered sufficient for diagnosis, while in all the other cases, at least one other tissue was analyzed, with skin fibroblast being considered the preferred tissue (11/25) (see Table 4).

Therefore, in case of suspected mT22, testing more than one tissue is highly recommended.

## 5. Suggested Recommendations for the Management of Suspected mT22 Cases

Prenatally, it might be important to consider the presence of an underlying aneuploidy when a IUGR fetus presents with dysmorphic ears and cardiac abnormalities. The other features to be investigated at the ultrasound scan might be hand anomalies or pelvis abnormalities, such as an anteriorized or atretic anus. Given the high variability of the fetal phenotype and the presence of pathological signs in various districts, a comprehensive anatomy assessment remains crucial. Based on our postnatal case review, it seems evident that many detectable phenotypical characteristics could already be identified in the prenatal period, even if they have not been described before in the literature. Such features were likely underreported in the past, as they were challenging to detect with the diagnostic tools available at that time. However, they represent a valid diagnostic opportunity in the present day. External ear anomalies can be better studied with 2D and 3D ultrasound scanning, as well as facial dysmorphisms. Anal abnormalities or genital malformations, such as hypospadias, can now be thoroughly studied with ultrasound scans. On the other hand, body asymmetry has been very frequently documented in postnatal life, while it has not been reported prenatally. This might be due to two main reasons: (A) the asymmetry becomes noticeable with postnatal growth only; (B) the common practice at the prenatal ultrasound scan leads the healthcare provider to measure only a single upper limb and a single lower limb, missing the opportunity to diagnose body asymmetry when only a first level scan is performed. In the hypothesis of an aneuploidy, invasive testing should be offered to establish the presence of an underlying mosaicism and rule out other genetically determined syndromes. Ideally, samples from both the placenta and amniotic fluid should be obtained, allowing for the best profiling possible for mosaic cases, but a personalized evaluation should be carried out for the effective need of multiple sampling, given the abortion risks connected to the invasive procedures [68]. The samples should be tested through traditional karyotyping and Array-CGH. Once mT22 is diagnosed, testing for uniparental disomy might help, even if imprinting is retained to be minimal on chromosome 22 according to the literature [63], and even if maternal UPD seems to have a poor impact on the phenotype [20,69]. It would be interesting to determine the UPD impact on IUGR for the two cases with maternal UPD described in this review, as UPD is a known cause of IUGR in the absence of placental mosaic [70]. In addition, in mT22 cases, serial growth scans should be scheduled in the second and third trimester, given the high risk of early and late IUGR reported in the literature. At the same time, maternal health should be carefully monitored, because of the maternal risk for preeclampsia reported in some prenatal mT22 cases and in pregnancies characterized by other types of placental mosaicism [71]. Furthermore, after birth, it is of great importance for future case reports to define the placenta’s characteristics. Additionally, in postnatal life, the literature describes features that might either go undetected prenatally, or be diagnosed postnatally only, such as neurodevelopmental delay, skin pigmentary changes, visual defects, or audiologic issues. From the prenatal literature review, it emerges that many patients with mT22 exhibit IUGR in prenatal life and are at higher risk of preterm birth. Therefore, when approaching the postnatal patient with suspected or proven mT22, it is important to consider that some patient features, such as failure to thrive, retarded growth pattern, or neurological sequelae, could be the result of fetal growth restrictions and/or prematurity [72,73].

Furthermore, postnatal evidence suggests that the presence of signs of chromosomal aneuploidy, even in the absence of intellectual disability, should not exclude the possibility of a diagnosis. Certainly, the prevalence of the conditions restricts the extent of a full phenotypic characterization. However, the conjoined presence of a congenital cardiovascular defect, body asymmetry, and pigmentary skin changes should raise a strong suspicion of chromosomal aneuploidy. Hence, since the first approach, evaluating the possibility of genetic analysis in different tissues is highly warranted. Moreover, especially in postnatal settings, it is important to keep in mind that composite phenotypes might derive from the co-occurrence of complex rearrangements in association with mT22. Such reports have been previously described in the literature [13,14,46,74], and a detailed description of such cases goes beyond the scope of this review.

In conclusion, based on evidence gathered so far, a few recommendations could be implemented to ensure more effective and personalized multidisciplinary management of individuals affected with mT22. In the postnatal setting, we suggest performing several comprehensive assessments, summarized in Table 5.

From a neuropsychological perspective, in the absence of a clear-cut, long-term follow-up for these patients in the literature, we consider it mandatory to initiate clinical follow-up as soon as possible [75]. Attention should be directed towards all aspects, ranging from intellectual functioning to adaptive abilities, including attention and higher-order functions such as executive functions, which, to date, have not been thoroughly investigated. There is also a need to further elucidate the behavioral aspects that play a significant role even in patients displaying normal intellectual functioning, as observed in our adolescent patient. More data are required to better delineate the neuropsychiatric phenotype, which appears to be both complex and sometimes subtle.

## 6. Conclusions

Mosaic trisomy 22 is rare in human life, and it is characterized by a wide range of signs and symptoms. At the prenatal level, fetuses frequently develop IUGR, and they might show facial and auricular dysmorphisms, heart defects, and extracardiac anomalies at the level of the hands and/or at the level of the urogenital system. The postnatal phenotype of the cases reported in literature is characterized by facial dysmorphism as well, such as frontal bossing, a flat nasal bridge, hypertelorism, ptosis, ear abnormalities, and a webbed neck. Evident postnatal clinical features are cardiac defects, skeletal defects, genitourinary malformations, pigmentary changes, and a variable degree of neurodevelopmental delay.

Therefore, we can conclude that prenatal and postnatal phenotypes are highly variable and may overlap with other aneuploidies or genetically-determined diseases. For this reason, a wise use of the analytic tools can help with the cytogenetic and molecular diagnosis. Knowledge of the potential postnatal characteristics and of the neurodevelopmental evolution can help healthcare professionals in the counseling of families and in the management of patients.

## Figures and Tables

**Table 1:** Summary of the prenatal case reports, their phenotypic characteristics collected at the prenatal ultrasound scan and at the post-mortem examination, their genetic profile, and the fate of the pregnancy.

	Sex	Phenotype	Mosaic	Fate
Schinzel et al., 1981 [3]	Female	Hypertelorism, retrognathia, low set ears with poorly formed auricles, anal atresia, tapering fingers, hypoplastic thumbs, partial cutaneous syndactyly (2nd and 3rd toes), VSD, ASD, RV hypoplasia, tricuspid atresia, ARSA, IUGR	Amniotic fluid 20%, Cord blood 4%, Blood 90%. No UPD	IUD 36 weeks
Stioui et al., 1989 [17]	Female	Normal morphology, late IUGR	Amniotic fluid 28–32%, CVS 100%. Blood, cord blood and skin: non-mosaic	Liveborn 33 weeks
Phillips et al., 1996 [18]	Male	Normal morphology, early IUGR	Placental mesenchymal cells 42%, Cytotrophoblast 40%, Kidney cells 4%; placental membranes, amniotic fluid, lungs, fibroblast from umbilical cord: non-mosaic. No UPD	TOP
De Pater et al., 1997 [9]	Female	Late IUGR, VSD, clinodactyly, micrognathia, low set ears	Amniotic fluid 20%, CVS 100%, skin 22%, blood: non-mosaic. Maternal UPD	Liveborn 39.6 weeks
Berghella et al., 1998 [19]	Male	Hypertelorism, bilateral pulmonary polylobation, large low set ears, choroid plexus cyst	Amniotic fluid 21%, skin 47%, lungs 3%, blood: non-mosaic	TOP
Bryan et al., 2002 [20]	Male	Early IUGR, oligohydramnios, hypospadias	CVS 100%, amniotic fluid and blood: non-mosaic. Maternal UPD	Liveborn 32 weeks
Wang et al., 2007 [21]	Female	IUGR, ASD, ductal aneurysm, hydrothorax, pericardial effusion, tricuspid regurgitation, LVNC, crumpled helix, low set ears, hypertelorism, tapered fingers	Amniotic fluid 35%, skin 76%, blood: non-mosaic	Liveborn 35.6 weeks
Leclercq et al., 2010 [1]	Female	Normal morphology	Amniotic fluid 16%, skin 6%, CVS and blood: non-mosaic	Liveborn
	Female	Dysmorphic ears, late IUGR	Amniotic fluid 14%	IUD 34 weeks
	Female	Nuchal fold thickening, hydrothorax	Amniotic fluid 26%, blood 4%	TOP
	Male	Early IUGR, multiple abnormalities	Blood 14%, amniotic fluid: non-mosaic	IUD 33 weeks
Mazza et al., 2010 [6]	Male	Late IUGR, low set ears, anteriorly displaced anus, pLSVC, ASD	Amniotic fluid 33%, CVS 95%, blood 33%, skin 4%, cord blood: non-mosaic. No UPD	Liveborn 37 weeks
Abdelgadir et al., 2013 [7]	Female	Tricuspid regurgitation, ASD, pericardial effusion, VNC. Hypertelorism, low set ears, dysmorphic left ear, thick nuchal fold, bilateral 5th finger clinodactyly, IUGR	Amniotic fluid 35%, skin 76%, blood: non-mosaic	Liveborn 35 weeks
	Female	TOF, thickened nuchal fold, short femur, single umbilical artery, bilateral 5th finger clino-brachydactyly, broad thumbs, anteriorly displaced anus, IUGR	Fibroblast from umbilical cord 50%, skin 3%, cardiomyocytes 17%, blood: non-mosaic	Liveborn 39 weeks
Chen et al., 2019 [22]	Female	Median facial cleft, oligohydramnios, IUGR, hypertelorism, low set ears	Amniotic fluid 50–60% skin 82%	TOP
Minella et al., 2023 [23]	Female	Pelvic kidney, IUGR	CVS 18%, amniotic fluid: non-mosaic	Liveborn 37 weeks
	Female	Early IUGR + Severe preeclampsia. Rhizomelic long bone shortening, hypertelorism, retrognathia, RV predominance, ARSA, low set ears, bilateral 5th finger clinodactyly	Amniotic fluid 3%, CVS 100%, kidney cells 14%	Perinatal death 24 weeks
	Female	Early IUGR, oligohydramnios, TOF, retrognathia, dysmorphic ears	Amniotic fluid (postnatal) 3%, blood 5%, CVS 15%	Perinatal death 24 weeks
Our cases	Female	Early IUGR, oligohydramnios, frontal bossing, flat nose, upslanting eyelids, wide mouth, micro-retrognathia, low set ears with dysmorphic helices, thickened nuchal fold, DORV, hypoplastic thymus, hands flexed, talipes, fixed limbs, single umbilical artery	Amniotic fluid 17%	Miscarriage 19 weeks
	Male	pLSVC, Late IUGR, nuchal fold thickening	Amniotic fluid 16%	Liveborn 33.6 weeks

Note: IUGR: Intrauterine growth restriction; ASD: Atrial septal defect; TOF: Tetralogy of Fallot; VSD: Ventricular septal defect; RV: Right ventricle; ARSA: Aberrant right subclavian artery; LVNC: Left ventricular non compaction; pLSVC: Persistent left superior vena cava; DORV: Double outlet right ventricle; CVS: Chorionic villi sampling; UPD: Uniparental disomy; TOP: Termination of pregnancy; IUD: Intra-uterine death.

**Table 2:** Summary of the most common prenatal findings in every district.

District	Cases on Total (n = 20)	Feature
No abnormalities	3	-
Systemic (17/20)	17	IUGR
	5	Nuchal fold thickening
	4	Oligohydramnios
Facial (10/20)	10	Low set ears
	7	Dysmorphic ears
	5	Hypertelorism
	6	Micro/Retrognathia
	1	Facial cleft
	1	Frontal bossing, flat nose, up-slanting eyelids, wide mouth
Cardiac (8/20)	4	ASD
	2	TOF
	2	VSD
	2	ARSA
	2	Tricuspid regurgitation
	2	Pericardial effusion
	2	VNC
	2	pLSVC
	1	RV hypoplasia
	1	RV predominance
	1	Tricuspid atresia
	1	Ductal aneurysm
	1	DORV
Hands (6/20)	4	Clinodactyly 5th fingers
	2	Tapered fingers
	1	Hypoplastic thumbs
	1	Broad thumbs
	1	Syndactyly
Urogenital (4/20)	2	Anteriorized anus
	1	Anal atresia
	1	Pelvic kidney
Miscellaneous	2	Short femur
	2	Hydrothorax
	3	Single umbilical artery
	1	Pulmonary polylobation
	1	Choroid plexus cyst
	1	Hypoplastic thymus, talipes, hands flexed, fixed limbs
	1	Abnormal unspecified

Note: IUGR: Intra-uterine growth restriction; ASD: Atrial septal defect; TOF: Tetralogy of Fallot; VSD: Ventricular septal defect; ARSA: Aberrant right subclavian artery; VNC: Ventricular non compaction; RV: Right ventricle; pLSVC: Persistent left superior vena cava; DORV Double Outlet Right Ventricle.

**Table 3:** Summary of the most common clinical findings in the postnatal cohort.

Clinical Features	Specific Feature	Cases on Total (n = 25)	Pt.1	Pt.2
Sex	F/M	15/10	M	M
Status at report	Alive/deceased	22/3	Alive	Alive
Growth pattern (15/25)	Delayed	15	−	−
	Failure to thrive	8	−	−
Facial Dysmorphisms (14/25)	Frontal bossing	8	−	−
	Microcephaly	6	−	−
	Hypertelorism	10	−	−
	Epicanthal folds	9	+	−
	Ptosis (mono-bilateral)	6	+	−
	Flattened nasal bridge	9	−	−
	Anteverted nostrils	5	−	−
	Long philtrum	5	−	−
	Thin lips	3	+	−
	Micro/retrognathia	6	−	−
	Low set ears	11	−	−
	Dysmorphic ears	10	+	−
	Pre-auricolar pits/tags	15	−	−
	Low posterior airline	7	−	−
	Cleft palate	3	−	−
	Webbed neck	7	+	−
Congenital heart disease (14/25)	Isolated defects	5	−	+
	Complex cardiac defect	5	−	−
	Combined cardiac and vascular defects	4	+	−
	ASD	7	+	−
	PVS	4	−	−
	VSD	6	−	−
	TOF	2	−	−
	PDA	3	−	−
Neurodevelopment (10/25)	Developmental delay	11	mild	no
	ID assessment	11	+	no
	Severe ID	1		
	Moderate ID	3		
	Mild ID	5		
	Normal–borderline ID	3	81 WISC−IV	
	Hypotonia	8	+	no
Audiological aspects (6/25)	Hearing loss	6	−	−
Skin and nails aspects (16/25)	Hypoplastic nails	12	−	−
	Skin Pigmentary changes	8	−	−
Skeletal (19/25)	Body asymmetry	11	+	−
	Craniofacial Symmetry	7	−	−
	Midface hypoplasia	6	+	−
	Radial anomalies	2	−	−
	5th finger Clinodactyly	11	+	−
	Syndactyly (partial/cutaneous)	6	−	−
	Radial anomalies	2	−	−
	5th finger Clinodactyly	11	+	−
	Syndactyly (partial/cutaneous)	6	−	−
	5th finger Clinodactyly	11	+	−
	Vertebral anomalies	4	+	−
Relevant anatomic anomalies (14/25)	G.i malrotation	2	−	−
	Anal malformation	4	+	−
	Genital anomalies	11	+	+
	Dental anomalies	6	+	−
	Palatal anomalies	4	−	−

Note: ASD: Atrial septal defect; PVS: Pulmonary Vein Stenosis; VSD: Ventricular septal defect; TOF: Tetralogy of Fallot; PDA: Patent Ductus Arteriosus; ID: Intellectual Disability.

**Table 4:** Summary of the DNA substrate for karyotyping analyzed in the postnatal cohort.

DNA Substrate for Karyotyping	N. of Procedures Reported	% of Cells Detected in the Analyzed Substrate
Amniotic epithelium	6	1–46%
Chorionic villi sampling	2	100% (CPM)
Lymphocytes from Cordocentesis	1	mT22 not detected
Fibroblasts from umbilical cord tissue	1	50%
Peripheral blood lymphocytes	9	1–68%
Skin fibroblasts	16	1.6–100 (SCM)%

Note: Abbreviations: CPM: Confined placental mosaicism; mT22: Mosaic trisomy 22; SCM: Skin-confined mosaicism.

**Table 5:** Suggested list of longitudinal evaluations and outpatient visits for the pediatric patient with mosaic trisomy 22.

Assessment	Aim
Genetic counseling	Discuss the genetic results, evaluate family history, and define recurrence risk.
Cardiovascular	Define potential cardiovascular defects by cardiological examination, including echocardiography and ECG.
ENT	Otorhinolaryngology consultation, along with auditory screening, to promptly detect hearing loss and palatal defects.
Ophthalmic	Assess presence of ophthalmic anomalies (ptosis, coloboma) and periodic evaluation of visual acuity.
Orthopedic	Assessment of potential scoliosis, vertebral anomalies, need for orthoses or surgical indication at time of diagnosis, and follow-up.
Dermatological	Estimate presence and extension of pigmentary changes.
General pediatric	Periodically, to assess general wellbeing, growth, and to evaluate the need of endocrinological investigations.
Surgical	Evaluation of surgical indication in case of detection of anatomic malformations (anal anomalies, external genital malformations).
Nc	Assess neurodevelopmental and behavioral aspects and intellectual functioning. Longitudinal observation is highly recommended.

## Data Availability

Data available upon request.

## References

[1] Leclercq S., Baron X., Jacquemont M., Cuillier F., Cartault F. (2010). Mosaic trisomy 22: Five new cases with variable outcomes. Implications for genetic counselling and clinical management. Prenat. Diagn..

[2] Martínez-Glez V., Tenorio J., Nevado J., Gordo G., Rodríguez-Laguna L., Feito M., Lapunzina P. (2020). A six-attribute classification of genetic mosaicism. Genet. Med..

[3] Schinzel A., Schmid W., Auf der Maur P., Moser H., Degenhardt K.H., Geisler M., Grubisic A. (1981). Incomplete trisomy 22. I. Familial 11/22 translocation with 3:1 meiotic disjunction. Delineation of a common clinical picture and report of nine new cases from six families. Hum. Genet..

[4] Sepulveda W., Be C., Schnapp C., Roy M., Wimalasundera R. (2003). Second-trimester sonographic findings in trisomy 22: Report of 3 cases and review of the literature. J. Ultrasound Med..

[5] Woods C.G., Bankier A., Curry J., Sheffield L.J., Slaney S.F., Smith K., Voullaire L., Wellesley D. (1994). Asymmetry and skin pigmentary anomalies in chromosome mosaicism. J. Med. Genet..

[6] Mazza V., Latella S., Fenu V., Ferrari P., Bonilauri C., Santucci S., Percesepe A. (2010). Prenatal diagnosis and postnatal follow-up of a child with mosaic trisomy 22 with several levels of mosaicism in different tissues. J. Obstet. Gynaecol. Res..

[7] Abdelgadir D., Nowaczyk M.J., Li C. (2013). Trisomy 22 Mosaicism and Normal Developmental Outcome: Report of Two Patients and Review of the Literature. Am. J. Med. Genet. Part A.

[8] Florez L., Lacassie Y. (2005). Mosaic trisomy 22: Report of a patient with normal intelligence. Am. J. Med. Genet. Part A.

[9] De Pater J.M., Schuring-Blom G.H., Bogaard R.V.D., Van Der Sijs-Bos C.J.M., Christiaens G.C.M.L., Stoutenbeek P., Leschot N.J. (1997). Maternal Uniparental Disomy for Chromosome 22 in a Child with Generalized Mosaicism for Trisomy 22. Prenat. Diagn..

[10] Page M.J., McKenzie J.E., Bossuyt P.M., Boutron I., Hoffmann T.C., Mulrow C.D., Shamseer L., Tetzlaff J.M., Akl E.A., Brennan S.E. (2021). The PRISMA 2020 statement: An updated guideline for reporting systematic reviews. BMJ Syst. Rev..

[11] National Library of Medicine (U.S.) (1999). National Library of Medicine Programs and Services. https://medlineplus.gov/.

[12] Wertelecki W., Breg W.R., Graham J.M., Iinuma K., Puck S.M., Sergovich F.R., Opitz J.M., Reynolds J.F. (1986). Trisomy 22 mosaicism syndrome and Ullrich-Turner stigmata. Am. J. Med. Genet..

[13] Naoufal R., Legendre M., Couet D., Gilbert-Dussardier B., Kitzis A., Bilan F., Harbuz R. (2016). Association of structural and numerical anomalies of chromosome 22 in a patient with syndromic intellectual disability. Eur. J. Med. Genet..

[14] Nardelli A., Laskoski L.V., Luiz A.F., Silveira M.A.D., D’arce L.P.G. (2023). Occurrence of mosaic trisomy 22 and pericentric inversion of chromosome 9 in a patient with a good prognosis. BMC Med. Genom..

[15] Dulitzky F., Shabtal F., Zlotogora J., Halbrecht I., Elian E. (1981). Unilateral radial aplasia and trisomy 22 mosaicism. J. Med. Genet..

[16] Osztovics M., Ivády G. (1977). A case of 22-trisomy mosaic. Acta Paediatr. Acad. Sci. Hung..

[17] Stioui S., de Silvestris M., Molinari A., Stripparo L., Ghisoni L., Simoni G. (1989). Trisomic 22 placenta in a case of severe intrauterine growth retardation. Prenat. Diagn..

[18] Phillips O.P., Tharapel A.T., Lerner J.L., Park V.M., Wachtel S.S., Shulman L.P. (1996). Risk of fetal mosaicism when placental mosaicism is diagnosed by chorionic villus sampling. Am. J. Obstet. Gynecol..

[19] Berghella V., Wapner R.J., Yang-Feng T., Mahoney M.J. (1998). Prenatal confirmation of true fetal trisomy 22 mosaicism by fetal skin biopsy following normal fetal blood sampling. Prenat. Diagn..

[20] Bryan J., Peters M., Pritchard G., Healey S., Payton D. (2002). A second case of intrauterine growth retardation and primary hypospadias associated with a trisomy 22 placenta but with biparental inheritance of chromosome 22 in the fetus. Prenat. Diagn..

[21] Wang J., Dang L., Mondal T.K., Khan A. (2007). Prenatally diagnosed mosaic trisomy 22 in a fetus with left ventricular non-compaction cardiomyopathy. Am. J. Med. Genet. Part A.

[22] Chen C.-P., Huang M.-C., Chern S.-R., Wu P.-S., Chen S.-W., Chuang T.-Y., Town D.-D., Wang W. (2019). Mosaic trisomy 22 at amniocentesis: Prenatal diagnosis and literature review. Taiwan. J. Obstet. Gynecol..

[23] Minella C., Jeandidier E., Koch A., Antal M.C., Favre R., Sananes N., Weingertner A.-S. (2023). Trisomy 22: First and Second Trimester Cytogenetic Analysis and Phenotypic Presentation in a Series of Seven Cases. Fetal Diagn. Ther..

[24] Cicero S., Bindra R., Rembouskos G., Spencer K., Nicolaides K.H. (2003). Integrated ultrasound and biochemical screening for trisomy 21 using fetal nuchal translucency, absent fetal nasal bone, free beta-hCG and PAPP-A at 11 to 14 weeks. Prenat. Diagn..

[25] Wright D., Kagan K.O., Molina F.S., Gazzoni A., Nicolaides K.H. (2008). A mixture model of nuchal translucency thickness in screening for chromosomal defects. Ultrasound Obstet. Gynecol..

[26] Hadlock F.P., Harrist R.B., Martinez-Poyer J. (1991). In utero analysis of fetal growth: A sonographic weight standard. Radiology.

[27] Bertino E., Spada E., Occhi L., Coscia A., Giuliani F., Gagliardi L., Gilli G., Bona G., Fabris C., De Curtis M. (2010). Neonatal Anthropometric Charts: The Italian Neonatal Study Compared with Other European Studies. J. Pediatr. Gastroenterol. Nutr..

[28] Growth Charts. 4 April 2023. https://www.cdc.gov/growthcharts/index.htm.

[29] Menasha J., Levy B., Hirschhorn K., Kardon N.B. (2005). Incidence and spectrum of chromosome abnormalities in spontaneous abortions: New insights from a 12-year study. Genet. Med..

[30] Kehinde F.I., Anderson C.E., McGowan J.E., Jethva R.N., Wahab M.A., Glick A.R., Sterner M.R., Pascasio J.M., Punnett H.H., Liu J. (2014). Co-occurrence of non-mosaic trisomy 22 and inherited balanced t(4;6)(q33;q23.3) in a liveborn female: Case report and review of the literature. Am. J. Med Genet. Part A.

[31] Eggenhuizen G.M., Go A., Koster M.P.H., Baart E.B., Galjaard R.J. (2021). Confined placental mosaicism and the association with pregnancy outcome and fetal growth: A review of the literature. Hum. Reprod. Update.

[32] Wright D., Syngelaki A., Bradbury I., Akolekar R., Nicolaides K. (2014). First-Trimester Screening for Trisomies 21, 18 and 13 by Ultrasound and Biochemical Testing. Fetal Diagn. Ther..

[33] Russo M.L., Blakemore K.J. (2014). A historical and practical review of first trimester aneuploidy screening. Semin. Fetal Neonatal Med..

[34] Nicolaides K.H., Azar G., Byrne D., Mansur C., Marks K. (1992). Fetal nuchal translucency: Ultrasound screening for chromosomal defects in first trimester of pregnancy. BMJ.

[35] Kelley J., McGillivray G., Meagher S., Hui L. (2021). Increased nuchal translucency after low-risk noninvasive prenatal testing: What should we tell prospective parents?. Prenat. Diagn..

[36] Paladini D., Donarini G., Bottelli L., Rossi A., Coltri A., Fulcheri E. (2021). Isolated, persisting, large choroid plexus cysts should warrant neurosonographic follow-up. Ultrasound Obstet. Gynecol..

[37] Schinzel A. (1981). Incomplete trisomy 22. III. Mosaic-trisomy 22 and the problem of full trisomy 22. Hum. Genet..

[38] Pridjian A.K., Frohlich E.D., VanMeter C.H., McFadden P.M., Ochsner J.L. (1995). Pharmacologic support with high-energy phosphate preservation in the postischemic neonatal heart. Ann. Thorac. Surg..

[39] Kalayinia S., Shahani T., Biglari A., Maleki M., Rokni-Zadeh H., Razavi Z., Mahdieh N. (2019). Mosaic trisomy 22 in a 4-year-old boy with congenital heart disease and general hypotrophy: A case report. J. Clin. Lab. Anal..

[40] Dayasiri K.C., De Silva D., Weerasekara K. (2018). Confirmation of mosaic trisomy 22 in an infant with failure to thrive. Sri Lanka J. Child Heal..

[41] Lin A.E., Santoro S., High F.A., Goldenberg P., Gutmark-Little I. (2020). Congenital heart defects associated with aneuploidy syndromes: New insights into familiar associations. Am. J. Med. Genet. Part C Semin. Med. Genet..

[42] Abuhamad A.Z., Chaoui R. (2012). A Practical Guide to Fetal Echocardiography: Normal and Abnormal Hearts.

[43] Patey O., Carvalho J.S., Thilaganathan B. (2019). Perinatal changes in cardiac geometry and function in growth-restricted fetuses at term. Ultrasound Obstet. Gynecol..

[44] Ruiter E.M., Toorman J., Hochstenbach R., de Vries B.B. (2004). Mosaic trisomy 22 in a boy with a terminal transverse limb reduction defect. Clin. Dysmorphol..

[45] Lessick M.L., Szego K., Wong P.W.K. (1988). Trisomy 22 Mosaicism with Normal Blood Chromosomes. Case report with literature review. Clin. Pediatr..

[46] Fruhman G., El-Hattab A.W., Belmont J.W., Patel A., Cheung S.W., Sutton V.R. (2011). Suspected trisomy 22: Modification, clarification, or confirmation of the diagnosis by aCGH. Am. J. Med. Genet. Part A.

[47] Pridjian G., Gill W.L., Shapira E. (1995). Goldenhar sequence and mosaic trisomy 22. Am. J. Med. Genet..

[48] Thomas S., Parker M., Tan J., Duckett D., Woodruff G. (2004). Ocular manifestations of mosaic trisomy 22: A case report and review of the literature. Ophthalmic Genet..

[49] Lund H.T., Tranebæerg L. (1990). Trisomy 22 Mosaicism Limited to Skin Fibroblasts in a Mentally Retarded, Dysmorphic Girl. Acta Paediatr. Scand..

[50] Crowe C.A., Schwartz S., Black C.J., Jaswaney V. (1997). Mosaic trisomy 22: A case presentation and literature review of trisomy 22 phenotypes. Am. J. Med. Genet..

[51] Allotey J., Lacaille F., Lees M.M., Strautnieks S., Thompson R.J., Davenport M. (2008). Congenital bile duct anomalies (biliary atresia) and chromosome 22 aneuploidy. J. Pediatr. Surg..

[52] Mollica F., Sorge G., Pavone L. (1977). Trisomy 22 mosaicism. J. Med. Genet..

[53] Basaran N., Berkil H., Ay N., Durak B., Ataman C., Ozdemir M., Ozon Y.H., Kaya I. (2001). A rare case: Mosaic trisomy 22. Ann. Genet..

[54] Hall T., Samuel M., Brain J. (2009). Mosaic trisomy 22 associated with total colonic aganglionosis and malrotation. J. Pediatr. Surg..

[55] Battle D.E. (2013). Diagnostic and Statistical Manual of Mental Disorders (DSM). Codas.

[56] Khan I., Leventhal B.L. (2023). Developmental Delay. StatPearls.

[57] Jansen S., Vissers L.E.L.M., de Vries B.B.A. (2023). The Genetics of Intellectual Disability. Brain Sci..

[58] Merks J.H., Ceelie N., Caron H.N., Hennekam R.C. (2004). Neuroblastoma, maternal valproic acid use, in-vitro fertilization and family history of mosaic chromosome 22: Coincidence or causal relationship?. Clin. Dysmorphol..

[59] Beedgen B., Querfeld U., Weiss-Wichert P., Nützenadel W. (1986). “Partial trisomy 22 and 11” due to a paternal 11;22 translocation associated with hirschsprung disease. Eur. J. Pediatr..

[60] dos Santos J.L., Quelhas P., Cerski C. (2022). Update on Etiology and Pathogenesis of Biliary Atresia. Curr. Pediatr. Rev..

[61] Gangbo E., Lacombe D., Alberti E.M., Taine L., Saura R., Carles D. (2004). Trisomy 22 with thyroid isthmus agenesis and absent gall bladder. Genet. Couns..

[62] Biesecker L.G., Spinner N.B. (2013). A genomic view of mosaicism and human disease. Nat. Rev. Genet..

[63] Ledbetter D., Engel E. (1995). Uniparental disomy in humans: Development of an imprinting map and its implications for prenatal diagnosis. Hum. Mol. Genet..

[64] Sdano M.R., Vanzo R.J., Martin M.M., Baldwin E.E., South S.T., Rope A.F., Allen W.P., Kearney H. (2014). Clinical Utility of Chromosomal Microarray Analysis of DNA from Buccal Cells: Detection of Mosaicism in Three Patients. J. Genet. Couns..

[65] Ballif B.C., Rorem E.A., Sundin K., Lincicum M., Gaskin S., Coppinger J., Kashork C.D., Shaffer L.G., Bejjani B.A. (2006). Detection of low-level mosaicism by array CGH in routine diagnostic specimens. Am. J. Med. Genet. Part A.

[66] Conlin L.K., Thiel B.D., Bonnemann C.G., Medne L., Ernst L.M., Zackai E.H., Deardorff M.A., Krantz I.D., Hakonarson H., Spinner N.B. (2010). Mechanisms of mosaicism, chimerism and uniparental disomy identified by single nucleotide polymorphism array analysis. Hum. Mol. Genet..

[67] Papavassiliou P., York T.P., Gursoy N., Hill G., Nicely L.V., Sundaram U., Jackson-Cook C. (2009). The phenotype of persons having mosaicism for trisomy 21/Down syndrome reflects the percentage of trisomic cells present in different tissues. Am. J. Med. Genet. A.

[68] Akolekar R., Beta J., Picciarelli G., Ogilvie C., D’Antonio F. (2015). Procedure-related risk of miscarriage following amniocentesis and chorionic villus sampling: A systematic review and meta-analysis. Ultrasound Obstet. Gynecol..

[69] Schinzel A.A., Basaran S., Bernasconi F., Karaman B., Yüksel-Apak M., Robinson W.P. (1994). Maternal uniparental disomy 22 has no impact on the phenotype. Am. J. Hum. Genet..

[70] Li M., Hao N., Jiang Y., Xue H., Dai Y., Wang M., Bai J., Lv Y., Qi Q., Zhou X. (2024). Contribution of uniparental disomy to fetal growth restriction: A whole-exome sequencing series in a prenatal setting. Sci. Rep..

[71] Yong P.J., Langlois S., von Dadelszen P., Robinson W. (2006). The association between preeclampsia and placental trisomy 16 mosaicism. Prenat. Diagn..

[72] Ream M.A., Lehwald L. (2018). Neurologic Consequences of Preterm Birth. Curr. Neurol. Neurosci. Rep..

[73] Smith A.E., Shah M., Badireddy M. (2023). Failure to Thrive. StatPearls.

[74] Heinrich T., Nanda I., Rehn M., Zollner U., Frieauff E., Wirbelauer J., Grimm T., Schmid M. (2012). Live-Born Trisomy 22: Patient Report and Review. Mol. Syndr..

[75] Romaniello R., Arrigoni F., De Salvo P., Bonaglia M.C., Panzeri E., Bassi M.T., Parazzini C., Righini A., Borgatti R. (2021). Long-term follow-up in a cohort of children with isolated corpus callosum agenesis at fetal MRI. Ann. Clin. Transl. Neurol..

